# Incidence and clinical outcomes of bacterial superinfections in critically ill patients with COVID-19

**DOI:** 10.3389/fmed.2023.1079721

**Published:** 2023-03-02

**Authors:** Si Mong Yoon, Jinwoo Lee, Sang-Min Lee, Hong Yeul Lee

**Affiliations:** ^1^Department of Critical Care Medicine, Seoul National University Hospital, Seoul, Republic of Korea; ^2^Division of Pulmonary and Critical Care Medicine, Department of Internal Medicine, Seoul National University Hospital, Seoul, Republic of Korea

**Keywords:** incidence, intensive care units, outcome, SARS-CoV-2, superinfection, COVID-19, thrombocytopenia

## Abstract

**Background:**

Bacterial superinfection is not uncommon in critically ill patients with coronavirus disease (COVID-19) pneumonia requiring intensive care unit (ICU) treatment. However, there is still a lack of evidence related to bacterial superinfection and their clinical significance in critically ill patients with COVID-19. Therefore, we assessed the incidence of bacterial superinfections and their effects on clinical outcomes in critically ill patients with COVID-19.

**Materials and methods:**

This single-center retrospective cohort study analyzed critically ill patients with COVID-19 admitted to the ICU at a tertiary academic hospital between February 2020 and December 2021. We reviewed data including patient demographics, clinical and microbiological characteristics, and outcomes.

**Results:**

During the study period, 106 patients (median [IQR] age, 67 [58–75] years) were included, of which 32 (30%) were diagnosed with bacterial superinfections. Of these, 12 cases (38%) were associated with multidrug-resistant pathogens. *Klebsiella aerogenes* (6 cases [19%]) and *Klebsiella pneumoniae* (6 cases [19%]) were the most common pathogens associated with superinfections. The median time to bacterial superinfection was 13 (IQR, 9–20) days after ICU admission. Patients with bacterial superinfections had significantly fewer ventilator-free days on day 28 (0 [IQR, 0–0] days) than those without bacterial superinfections (19 [IQR, 0–22] days) (*p* < 0.001). Patients with bacterial superinfections had a longer ICU length of stay (32 [IQR, 9–53] days) than those without bacterial superinfections (11 [IQR, 7–18] days) (*p* < 0.001). Additionally, they had a longer hospital length of stay after ICU admission (39 [IQR, 18–62] days) than those without bacterial superinfections (18 [IQR, 12–37] days) (*p* = 0.001). There were no differences in ICU mortality or in-hospital mortality between the two groups. In the multivariable analysis, higher SAPS II score (OR, 2.697; 95% CI, 1.086–6.695) and thrombocytopenia (OR, 3.318; 95% CI, 1.355–8.123) were identified as risk factors for development of bacterial superinfection.

**Conclusion:**

In critically ill patients with COVID-19, bacterial superinfections were common, and more than one-third of the bacterial superinfection cases were caused by multidrug-resistant pathogens. As patients with bacterial superinfections had worse clinical outcomes, the development of bacterial superinfections should be actively monitored.

## 1. Introduction

The severe acute respiratory syndrome coronavirus-2 (SARS-CoV-2) infection pandemic has lasted for more than 2 years since 2020. Over 500 million confirmed cases have been reported worldwide, and over six million deaths have been recorded (1). Because the SARS-CoV-2 infection often leads to the development of acute respiratory distress syndrome (ARDS), critically ill patients with severe coronavirus disease (COVID-19) pneumonia often require prolonged mechanical ventilation (2–4). One recent randomized controlled trial showed that early administration of dexamethasone in ARDS reduced the duration of mechanical ventilation and overall mortality (5). Moreover, the use of dexamethasone in COVID-19 ARDS has become commonplace, as it was found to lower mortality risk in patients requiring oxygen therapy and mechanical ventilation (6). Unfortunately, however, a study conducted by Bernard et al. (7) showed that the use of high-dose glucocorticoids was associated with an increased risk of secondary bacterial infections in patients with ARDS. In addition, the SARS-CoV-2 has been reported to cause immune dysregulation through increase of neutrophil-lymphocyte-ratio, and T lymphopenia (8). Thus, with the increased use of glucocorticoids compounded with a dysregulated host immune response caused by the SARS-CoV-2, secondary infections in critically ill patients with COVID-19 have become a major concern.

Studies of previous viral pandemics have showed that additional bacterial infections were associated with increased mortality, higher rates of respiratory distress, and more frequent ICU admissions (9, 10). As such, a better understanding of bacterial superinfections in COVID-19 is needed to improve patient outcomes. However, current literature on bacterial superinfections in COVID-19 is scarce, and most studies have focused on patients with mild-to-moderate illness severity rather than critically ill patients admitted to the ICU (11–15). Although the current guidelines recommend empirical therapy with antimicrobials in patients with severe COVID-19 pneumonia, there is a lack of evidence related to bacterial superinfection including multidrug-resistant (MDR) pathogen infections, and their clinical significance (16, 17).

In this study, we hypothesized that critically ill patients with COVID-19 who develop bacterial superinfection are at increased risk for worse clinical outcomes. Here, we assessed the incidence of bacterial superinfection, including new episodes of pneumonia, urinary tract infection (UTI), bacteremia, or other infections, and those of multidrug-resistant pathogens and determined their effects on clinical outcomes in critically ill patients with severe COVID-19 pneumonia who require ICU admission.

## 2. Materials and methods

### 2.1. Study design and patients

This single-center, retrospective cohort study analyzed critically ill patients with severe COVID-19 pneumonia who were admitted to a 12-bed disaster ICU at Seoul National University Hospital, a tertiary academic hospital in South Korea that served as a nationally designated hospital for patients with severe and critical COVID-19, between February 2020 and December 2021.

Adult patients (aged ≥18 years) who were diagnosed with COVID-19 through reverse transcription-polymerase chain reaction assay and were admitted to the disaster ICU due to severe COVID-19 pneumonia were included and followed up until the time of hospital discharge or death. According to the World Health Organization guidelines for COVID-19, severe COVID-19 pneumonia was defined as the presence of at least one of the following: oxygen saturation < 90% in room air or signs of severe respiratory distress (accessory muscle use, inability to complete full sentences, or respiratory rate > 30 breaths per minute) (13). We excluded patients who had confirmed bacterial infections within 6 months prior to ICU admission or within 48 h after ICU admission, completed a Physician Orders for Life-Sustaining Treatment (POLST, including do-not-intubate orders) form, stayed in the ICU for less than 48 h, or were transferred from an overseas hospital. The Institutional Review Board (IRB) of Seoul National University Hospital waived the requirement for written informed consent and approved this study (approval number: IRB-H-2106-213-1,231).

### 2.2. Data collection

We reviewed the following data of all the patients in our database: demographic characteristics, comorbidities, Charlson comorbidity index, Acute Physiology and Chronic Health Evaluation II (APACHE II) score, Sequential Organ Failure Assessment (SOFA) score, Simplified Acute Physiology Score II (SAPS II) score, site of sample collection and method (sputum culture, endotracheal aspirates, bronchoscopic washing, bronchoalveolar lavage, urine culture, blood culture, or other cultures), bacterial species, antibiotic susceptibility, and clinical outcomes. During the study period, all patients included in this study underwent systematic screening for colonization by MDR bacteria (nasal methicillin-resistant *Staphylococcus aureus*, sputum carbapenem-resistant *Acinetobacter baumannii*, rectal vancomycin-resistant *Enterococci*, and rectal carbapenem-resistant *Enterobacteriaceae*) on ICU admission. These data were also reviewed. Based on the definition used in previous studies, an immunocompromised condition was defined as a diagnosis of primary immunodeficiency disorder, a diagnosis of human immunodeficiency virus (HIV) infection or acquired immune deficiency syndrome (AIDS), solid organ/hematopoietic stem cell transplant recipients, and receipt of any chemotherapy or immunosuppressants, including corticosteroids (prednisolone ≥20 mg/day, or an equivalent dose of other corticosteroids, for 2 weeks or longer) in the 6 months prior to COVID-19 diagnosis (18–20). Although the definition of ARDS under high-flow nasal oxygen (HFNO) is unclear and there is a difference in the PaO_2_:FiO_2_ ratio compared to when the mechanical ventilator is applied (21), we used an expanded definition of ARDS as follows (22): PaO_2_:FiO_2_ ratio ≤ 300 mmHg, patients treated with HFNO of at least 30 L/min or with a positive end-expiratory pressure ≥ 5 cm of water, and bilateral infiltrates documented by chest radiography or a computed tomography scan.

### 2.3. Bacterial superinfection and multidrug-resistant pathogens

A blood culture was obtained within 24 h of ICU admission, and thereafter, microbiologic samples were obtained according to the discretion of the attending intensivists. Bacterial superinfection was defined as clinical deterioration and the presence of bacteria identified in the lower respiratory tract (sputum, endotracheal aspirates, bronchoscopic washing, or bronchoalveolar lavage) urine culture, blood culture, or other culture samples (e.g., pleural effusion, ascitic fluid) after 48 h of ICU admission. MDR pathogens were defined as bacteria that are resistant to three or more types (one or more of each type) of antibiotics with different structures (different mechanisms of action) (23, 24).

### 2.4. Statistical analysis

Continuous variables were reported as medians and interquartile ranges (IQR), and categorical variables were expressed as counts and percentages. Between-group differences in baseline characteristics were assessed using the chi-square test or Fisher’s exact test for qualitative variables and Student *t*-test or Mann–Whitney *U* test for quantitative variables. Univariable and multivariable logistic regression analyses were used to identify the risk factors for bacterial superinfection in patients with severe COVID-19 pneumonia. Independent variables were selected based on biological plausibility and associations in the scientific literature (15, 25, 26). All the variables with a *p-*value of <0.20 in the univariable analysis were included in the multivariable stepwise backward logistic regression model to avoid model overfitting (27). In addition, we generated a receiver-operating characteristic (ROC) curve and estimated the area under the curve (AUROC) to determine the predictive value and optimal cut-off values of variables with a value of p of less than 0.05 in the multivariable logistic regression analysis of risk factors for the development of bacterial superinfection. The optimal cut-off values were determined based on Youden’s index, which maximizes the sum of the sensitivity and specificity. The results were presented as odds ratios (OR) with 95% confidence intervals (CI). All the analyses were two-tailed, and *p*-values less than 0.05 were considered significant. IBM SPSS Statistics (version 25.0 for Windows; IBM, Armonk, NY, United States) was used for all the statistical analyses.

## 3. Results

### 3.1. Patient characteristics

**Figure 1:**
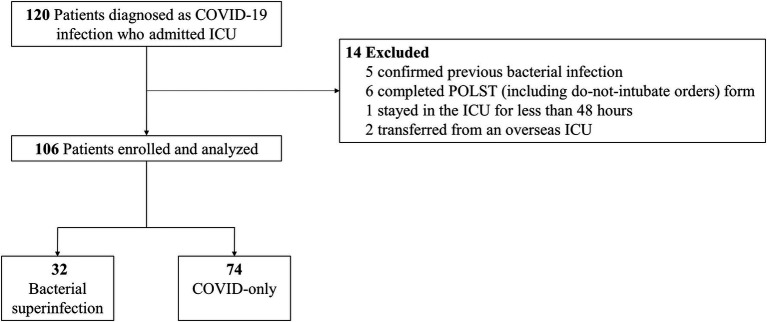
Flow diagram of the study population. POLST, portable orders for life-sustaining treatment; ICU, intensive care unit; COVID-19, coronavirus disease.

Of the 120 patients assessed for eligibility, 14 patients were excluded for the following reasons: (1) five patients had confirmed bacterial infections within 6 months prior to ICU admission or within 48 h after ICU admission, (2) six patients completed a POLST (including do-not-intubate orders) form, (3) one patient stayed in the ICU for less than 48 h, and (4) two patients were transferred from an overseas ICU. A total of 106 patients were included in this study, of which 32 (30%) were diagnosed with bacterial superinfections (bacterial superinfection group), and 74 (70%) were without bacterial superinfections (COVID-only group) (Figure 1).

Table 1 shows the demographic and clinical characteristics at the baseline. The median age was 67 (IQR, 58–75) years, 65% of the patients were men, the median body mass index was 24.2 (IQR, 22.6–25.8) kg/m^2^, 52% had hypertension, and 34% had diabetes mellitus. The demographic and clinical characteristics of the patients at baseline were comparable between the two groups, except for the platelet count, SOFA score, and SAPS II score. The bacterial superinfection group had significantly lower platelet counts (*p* = 0.005) and higher SOFA (*p* = 0.008) and SAPS II scores (*p* = 0.011) than those of the COVID-only group.

**Table 1:** Baseline and clinical characteristics of critically ill patients with COVID-19.

	Total	Bacterial superinfection	COVID-only	*p*–value
Variables	(*n* = 106)	(*n* = 32)	(*n* = 74)	
Age, years	67 (58–75)	67 (61–72)	68 (58–76)	0.508
Male, *n* (%)	69 (65)	24 (75)	45 (61)	0.159
BMI, kg/m^2^	24.2 (22.6–25.8)	24.3 (22.7–27.1)	24.2 (22.6–25.5)	0.529
Comorbidities, *n* (%)				
Hypertension	55 (52)	14 (44)	41(55)	0.270
Diabetes mellitus	36 (34)	11 (34)	25 (34)	0.953
Heart failure	1 (1)	0 (0)	1 (1)	>0.999
Chronic liver disease	1 (1)	0 (0)	1 (1)	>0.999
Chronic kidney disease	11 (10)	4 (13)	7 (10)	0.637
Chronic obstructive pulmonary disease	5 (5)	3 (9)	2 (3)	0.160
Immunocompromised	7 (7)	2 (6)	5 (7)	>0.999
Charlson comorbidity index	3 (2–4)	3 (2–4)	3 (2–4)	0.735
Upon ICU admission				
APACHE II score	13 (10–21)	17 (11–22)	12 (9–20)	0.099
SOFA score	5 (3–10)	9 (5–12)	4 (3–8)	0.008
SAPS II score	33 (23–46)	40 (28–57)	31 (21–41)	0.011
Screening test, *n* (%)				
Nasal MRSA	3 (3)	2 (6)	1 (1)	0.203
Sputum CRAB	0 (0)	0 (0)	0 (0)	
Rectal VRE	4 (4)	1 (3)	3 (4)	>0.999
Rectal CRE	2 (2)	0 (0)	2 (3)	>0.999
Laboratory data				
WBC, 10^3^/μL	7.9 (5.4–10.7)	8.3 (6.5–12.7)	7.4 (5.3–10.3)	0.366
Leukocytes count, 10^3^/μL	6.9 (4.5–9.2)	7.4 (5.9–11.0)	6.4 (4.4–8.8)	0.396
Lymphocytes count, 10^3^/μL	0.7 (0.5–0.9)	0.6 (0.3–0.9)	0.6 (0.4–0.8)	0.981
Platelets count, 10^3^/μL	195 (134–255)	135 (98–193)	220 (149–276)	0.005
Fibrinogen, mg/dL	436 (354–502)	432 (323–489)	447 (364–518)	0.069
Lactic acid, mmol/L	1.4 (1.1–1.9)	1.4 (1.2–1.9)	1.4 (1.0–2.0)	0.547
CRP, mg/dL	7.9 (4.2–17.3)	9.4 (3.7–18.3)	7.4 (4.2–15.8)	0.575
Procalcitonin, ng/mL	0.2 (0.1–0.5)	0.2 (0.1–0.7)	0.2 (0.1–0.5)	0.918
LDH, IU/L	478 (346–593)	494 (349–608)	472 (341–569)	0.890

Values are presented as number (%), median (interquartile range). LOS, length of stay; ICU, intensive care unit; APACHE II, Acute Physiology and Chronic Health Evaluation II; SOFA, Sequential Organ Failure Assessment; SAPS II, Simplified Acute Physiology Score II; MRSA, methicillin-resistant *Staphylococcus aureus*; CRAB, carbapenem-resistant *Acinetobacter baumannii*; VRE, vancomycin-resistant *Enterococci*; CRE, carbapenem-resistant Enterobacteriaceae; CRP, C-reactive protein; LDH, lactate dehydrogenase.

### 3.2. Interventions and clinical outcomes

Table 2 summarizes the interventions required during ICU stay and the clinical outcomes according to the presence of bacterial superinfection. ARDS was identified in 89 of 106 (84%) critically ill patients with severe COVID-19 pneumonia: 91% (29 of 32 patients) in the bacterial superinfection group and 81% (60 of 74 patients) in the COVID-only group (*p* = 0.219). There were no significant differences in the interventions required during ICU stay between the two groups, such as HFNO, mechanical ventilator, extracorporeal membrane oxygenator, renal replacement therapy, prone positioning, nitric oxide use, and vasopressor use.

**Table 2:** Interventions and clinical outcomes in critically ill patients with COVID-19.

	Total	Bacterial superinfection	COVID-only	*p*-value
Variables	(*n* = 106)	(*n* = 32)	(*n* = 74)	
Acute respiratory distress syndrome, *n* (%)^*^	89 (84)	29 (91)	60 (81)	0.219
Life support treatment during ICU stay, *n* (%)				
High-flow nasal oxygen	101 (95)	30 (94)	71 (96)	0.637
Mechanical ventilation	78 (74)	27 (84)	51 (69)	0.098
Extracorporeal membrane oxygenator	8 (8)	4 (13)	4 (5)	0.239
Renal replacement therapy	12 (11)	6 (19)	6 (8)	0.178
Prone positioning	74 (70)	24 (75)	50 (68)	0.444
Inhaled nitric oxide	16 (15)	8 (25)	8 (11)	0.078
Vasopressor	80 (76)	28 (88)	52 (70)	0.058
Clinical outcomes				
Hospital length of stay after ICU admission, days (IQR)	22 (13–45)	39 (18–62)	18 (12–37)	0.001
ICU length of stay, days (IQR)	12 (7–32)	32 (9–53)	11 (7–18)	<0.001
Ventilator-free days at day 28, days (IQR)^**^	15 (0–21)	0 (0–0)	19 (0–22)	<0.001
ICU mortality, *n* (%)	20 (19)	7 (22)	13 (18)	0.603
In-hospital mortality, *n* (%)	22 (21)	8 (25)	14 (19)	0.478
28-day mortality, *n* (%)	9 (9)	4 (13)	5 (7)	0.446

^*^Acute respiratory distress syndrome definition: (1) PaO_2_:FiO_2_ ratio ≤ 300 mmHg, (2) patients treated with HFNO of at least 30 l/min or with a positive end-expiratory pressure ≥ 5 cm of water, and (3) bilateral infiltrates documented by chest radiography or computed tomography scan. ^**^Calculated for patients receiving mechanical ventilator treatment (27 for bacterial superinfection group and 51 for COVID-only group). ICU, intensive care unit, IQR, interquartile range.

The median time to bacterial superinfection was 13 (IQR, 9–20) days after ICU admission. Among the patients who received mechanical ventilator treatment during ICU stay (27 [84%] patients in the bacterial superinfection group and 51 [69%] patients in the COVID-only group), ventilator-free days at 28 days were significantly lower in the bacterial superinfection group than those in the COVID-only group: 0 (IQR, 0–0) days versus 19 (IQR, 0–22) days (*p* < 0.001) (Table 2). Moreover, the ICU length of stay was significantly longer in the bacterial superinfection group than that in the COVID-only group: 32 (IQR, 9–53) days versus 11 (IQR, 7–18) days (*p* < 0.001). Additionally, the length of hospital stay after ICU admission was significantly longer in the bacterial superinfection group than that in the COVID-only group: 39 (IQR, 18–62) days versus 18 (IQR, 12–37) days (*p* = 0.001). ICU mortality, in-hospital mortality, and 28-day mortality were higher in the bacterial superinfection group, but these differences were not statistically significant.

### 3.3. Microbiological results and risk factors of bacterial superinfection

**Figure 2:**
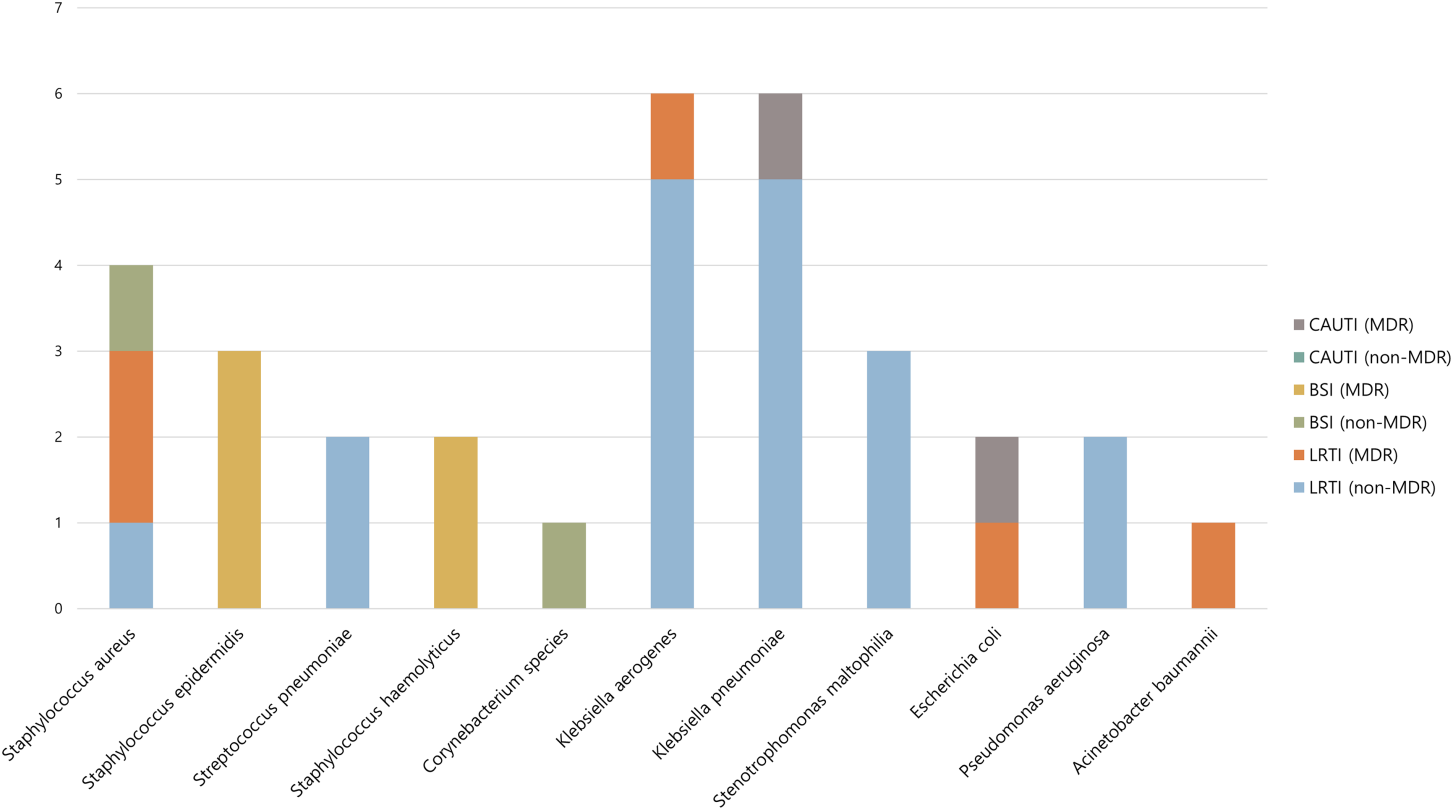
Causative microorganism of bacterial superinfection according to site of infection and MDR status. MDR, multidrug-resistant; CAUTI, catheter-associated urinary tract infection; BSI, bloodstream infection; LRTI, lower respiratory tract infection.

Of the 32 bacterial superinfections, 12 (38%) were caused by MDR pathogens. Gram-positive and Gram-negative bacteria were responsible for 12 and 20 cases, respectively, of superinfection, of which 7 (58%) and 5 (25%), respectively, were caused by MDR pathogens. Table 3 shows the types of bacteria that caused bacterial superinfections and the MDR status according to the source of infection. Of the 23 cases of lower respiratory tract infections, 5 (22%) were caused by MDR pathogens. Of the 7 cases of bloodstream infections, 5 (71%) were caused by MDR pathogens. Catheter-associated urinary tract infections were caused only by MDR pathogens. The most common pathogens associated with bacterial superinfections were *Klebsiella aerogenes* (6 cases [19%]) and *Klebsiella pneumoniae* (6 cases [19%]) (Figure 2).

**Table 3:** Source of superinfection and microbiology in critically ill patients with COVID-19.

Bacteria		Lower respiratory tract infection		Bloodstream infection		Catheter-associated urinary tract infection		Total
		Non-MDR	MDR	Non-MDR	MDR	Non-MDR	MDR	
G(+)	*Staphylococcus aureus*	1	2	1	0	0	0	4
	*Staphylococcus epidermidis*	0	0	0	3	0	0	3
	*Streptococcus pneumoniae*	2	0	0	0	0	0	2
	*Staphylococcus haemolyticus*	0	0	0	2	0	0	2
	*Corynebacterium* species	0	0	1	0	0	0	1
G(−)	*Klebsiella aerogenes*	5	1	0	0	0	0	6
	*Klebsiella pneumoniae*	5	0	0	0	0	1	6
	*Stenotrophomonas maltophilia*	3	0	0	0	0	0	3
	*Escherichia coli*	0	1	0	0	0	1	2
	*Pseudomonas aeruginosa*	2	0	0	0	0	0	2
	*Acinetobacter baumannii*	0	1	0	0	0	0	1
		18	5	2	5	0	2	32

MDR, multidrug-resistant; G(+), gram positive; G(−), gram negative.

In the univariable analysis, a high SOFA score (OR, 1.132; 95% CI, 1.022–1.254; *p* = 0.018), or SAPS II score (OR, 1.031; 95% CI, 1.006–1.056; *p* = 0.016), and a low platelet count (OR, 0.992; 95% CI, 0.987–0.998; *p* = 0.006) were identified as risk factors for the development of bacterial superinfection (Table 4). In the multivariable analysis model 1, platelet count, APACHE II, and SAPS II scores were treated as continuous variables. The SAPS II score (OR, 1.065; 95% CI, 1.013–1.121; *p* = 0.014) and platelet count (OR, 0.993; 95% CI, 0.987–0.998; *p* = 0.011) were identified as risk factors for the development of bacterial superinfection (Table 4). We generated an ROC curve and estimated the AUROC to determine the predictive value and optimal cut-off value of SAPS II and platelet count for the development of bacterial superinfection. Using a cut-off value of 42, the SAPS II score predicted bacterial superinfection with a sensitivity of 50.0% and specificity of 75.7% with an AUROC of 0.647 (95% CI, 0.536–0.759; *p* < 0.001). Using a cut-off value of 172,000/μL, platelet count predicted bacterial superinfection with a sensitivity of 71.9% and specificity of 68.9% with an AUROC of 0.696 (95% CI, 0.577–0.816; p < 0.001). In the multivariable analysis model 2, platelet count, APACHE II, and SAPS II scores were treated as categorical variables. Based on the results of the ROC curve analysis and biological plausibility, a SAPS II score of 42, an APACHE II score of 11, and a platelet count of 150,000/μL were set as the cut-off values in the present study. The SAPS II score (OR, 2.697; 95% CI, 1.086–6.695; *p* = 0.032) and platelet count (OR, 3.318; 95% CI, 1.355–8.123; *p* = 0.012) were identified as risk factors for the development of bacterial superinfection.

**Table 4:** Risk factors for the development of bacterial superinfection in critically ill patients with COVID-19.

	Univariable		Model 1^*^		Model 2^**^	
	OR (95% CI)	*p*-value	OR (95% CI)	*p*-value	OR (95% CI)	*p*-value
Age	1.011 (0.979–1.044)	0.504				
Male sex	1.933 (0.766–4.882)	0.163				
Charlson comorbidity index	1.041 (0.827–1.310)	0.907				
Immunocompromised	0.920 (0.169–5.010)	0.923				
APACHE II score	1.046 (0.991–1.103)	0.103	0.912 (0.817–1.018)	0.100		
SOFA score	1.132 (1.022–1.254)	0.018				
SAPS II score	1.031 (1.006–1.056)	0.016	1.065 (1.013–1.121)	0.014	2.697 (1.086–6.695)	0.032
Neutrophil count	1.000 (1.000–1.000)	0.395				
Lymphocyte count	1.000 (0.999–1.001)	0.981				
Platelet count	0.992 (0.987–0.998)	0.006	0.993 (0.987–0.998)	0.011	3.318 (1.355–8.123)	0.012
Fibrinogen	0.996 (0.992–1.000)	0.073				

^*^In model 1, platelet count, APACHE II, and SAPS II scores were treated as continuous variables. All the variables with a *p-*value of <0.20 in the univariable analysis were included in the multivariable stepwise backward logistic regression model. ^**^In model 2, APACHE II score (APACHE II ≥11), SAPS II score (SAPS II ≥42), and platelet count (platelet count <150,000/mm^3^) were treated as categorical variables. All the variables with a *p-*value of <0.20 in the univariable analysis were included in the multivariable stepwise backward logistic regression model. OR, odds ratio; APACHE II, Acute Physiology and Chronic Health Evaluation II; SOFA, Sequential Organ Failure Assessment; SAPS II, Simplified Acute Physiology Score II.

### 3.4. Thrombocytopenia and clinical outcomes

Further sensitivity analysis was performed by dividing the patients into two groups according to their risk factors for the development of bacterial superinfection: a high-risk group with a SAPS II score ≥ 42 or thrombocytopenia (platelet count <150,000/μL), and a low-risk group with a SAPS II score < 42 and without thrombocytopenia (Table 5). The incidence of bacterial superinfection was significantly higher in the high-risk group than in the low-risk group: 49.1% (26 of 53 patients) versus 11.3% (6 of 53 patients), respectively (*p* < 0.001). Of the 32 cases of bacterial superinfection, 12 (38%) were caused by MDR pathogens and were observed only in the high-risk group. Among the patients who received mechanical ventilator treatment during their ICU stay (45 [85%] patients in the high-risk group and 33 [62%] patients in the low-risk group), the number of ventilator-free days at day 28 were significantly lower in the high-risk group than in the low-risk group: 0 (IQR, 0–18) days versus 21 (IQR, 18–23) days (*p* < 0.001). Moreover, the length of ICU stay was significantly longer in the high-risk group than in the low-risk group: 24 (IQR, 10–46) days versus 10 (IQR, 5–14) days (*p* < 0.001). Additionally, the length of hospital stay after ICU admission was significantly longer in the high-risk group than in the low-risk group: 33 (IQR, 15–64) days versus 16 (IQR, 11–28) days (*p* < 0.001). The ICU mortality, in-hospital mortality, and 28-day mortality rates were significantly higher in the high-risk group than in the low-risk group (Table 5).

**Table 5:** Bacterial superinfection and clinical outcomes in critically ill patients with COVID-19 according to their SAPS II score and platelet count.

	High-risk^*^	Low-risk^*^	*p*-value
	(*n* = 53)	(*n* = 53)	
Bacterial superinfection, *n* (%)	26 (49)	6 (11)	<0.001
MDR pathogen superinfection, *n* (%)	12 (23)	0 (0)	<0.001
Treatment outcomes			
Hospital length of stay after ICU admission, days (IQR)	33 (15–64)	16 (11–28)	<0.001
ICU length of stay, days (IQR)	24 (10–46)	10 (5–14)	<0.001
Ventilator-free days at day 28, days (IQR)^**^	0 (0–18)	21 (18–23)	<0.001
ICU mortality, *n* (%)	16 (30)	4 (8)	0.003
In-hospital mortality, *n* (%)	18 (34)	4 (8)	0.001
28-day mortality, *n* (%)	8 (15)	1 (2)	0.015

^*^The high-risk group was defined as patients with SAPS II ≥42 or thrombocytopenia (platelets <150,000/μL), and the low-risk group was defined as patients without both conditions. ^**^Calculated for patients receiving mechanical ventilator treatment (45 for high-risk group and 33 for low-risk group). COVID-19, coronavirus disease; SAPS II, Simplified Acute Physiology Score II; MDR, multidrug-resistant; ICU, intensive care unit; IQR, interquartile range.

## 4. Discussion

In this retrospective cohort study of critically ill patients with severe COVID-19 pneumonia, there were 32 cases of infections with bacterial pathogens, newly confirmed after ICU admission. More than one-third of the cases were associated with MDR pathogens, with *Klebsiella aerogenes* and *Klebsiella pneumoniae* being the most common pathogens. Patients with bacterial superinfections had worse clinical outcomes, including fewer ventilator-free days, longer ICU stay, and longer hospital stay after ICU admission. However, there were no statically significant differences in ICU and in-hospital mortality between patients with and without bacterial superinfections. A higher SAPS II score and thrombocytopenia were independent risk factors for the development of bacterial superinfection. Moreover, patients with such risk factors had a significantly higher incidence of bacterial superinfection and worse clinical outcomes than those without risk factors. Notably, bacterial superinfection caused by MDR pathogens occurred only in patients with risk factors.

Our results are consistent with those of previous studies that reported the bacterial superinfection rate as 9–59% (11, 12, 15, 25, 26, 28, 29). Of these, three studies were conducted on ICU patients, and it is judged that the incidence rate has varied due to differences in the definition of superinfection. A study conducted in Iran used only sputum and tracheal aspirates without bronchoscopic examination when acquiring respiratory specimens, and reported a superinfection incidence rate of 12% (29). In the study conducted in Spain, the incidence rate of superinfection was reported as 41% including fungal superinfection (25). Finally, a study conducted in the United States reported a ventilator-associated pneumonia incidence rate of 44% using multiplex PCR in addition to quantitative culture with bronchoalveolar lavage specimens (28). In particular, Bardi et al. (25) reported that the median time to superinfection was 9 (IQR, 5–11) days and Pickens et al. (28) reported that the average time to ventilator-associated pneumonia was 10.8 days, which are similar to our results. Therefore, the development of bacterial superinfections should be carefully monitored in critically ill patients with severe COVID-19 pneumonia who require hospitalization for more than 1 week.

In the present study, patients with bacterial superinfections had significantly fewer ventilator-free days than those without bacterial superinfections. Moreover, as the liberation from mechanical ventilation was delayed, the ICU length of stay was significantly greater in patients with bacterial superinfections than in those without bacterial superinfections. Additionally, the aforementioned study by Bardi et al. (25) reported that bacterial superinfections in the ICU were associated with an increase in ICU length of stay and mortality. Although our results were not statistically significant, ICU mortality was numerically higher in the patients with bacterial superinfections than that in those without bacterial superinfections. Our study may have been underpowered to detect a clinically important difference in mortality. To date, studies analyzing the risk of bacterial superinfections in critically ill patients with severe COVID-19 pneumonia admitted to the ICU are rare. Previous studies have reported that low lymphocyte count at baseline, diabetes, APACHE II score, use of interleukin-6 receptor antagonists, use of corticosteroids, and ICU length of stay were risk factors for the development of bacterial superinfections (15, 25, 26). In the univariable analysis of our study, SOFA score, SAPS II score, and platelet count were found to be risk factors for the development of bacterial superinfection. In the multivariable analysis, the SAPS II score and platelet count were identified as independent risk factors, regardless of whether these variables were treated as categorical or continuous variables in the analysis. Moreover, patients with such risk factors had a significantly higher incidence of bacterial superinfection and worse clinical outcomes than those without risk factors. The results of this study suggest that thrombocytopenia and high SAPS II score are associated with an increased risk of bacterial superinfection and worse clinical outcomes.

There are several possible mechanisms whereby thrombocytopenia may occur in patients with COVID-19. First, SARS-CoV-2 can directly infect bone marrow, which may reduce platelet production (30, 31). Second, megakaryocytes dynamically release platelets during pulmonary circulation (32), and in patients with lung consolidation due to COVID-19 pneumonia, the damaged pulmonary capillary bed causes megakaryocyte rupture and prevents platelet release. Third, damaged lung tissue and pulmonary endothelial cells activate platelets in lung tissue and increase platelet consumption by creating microthrombi (30). In addition, thrombocytopenia can occur in patients with COVID-19 for various other reasons such as decreased thrombopoietin (TPO) production as a result of parenchymal liver injury, immune thrombocytopenic purpura (ITP), heparin-induced thrombocytopenia (HIT), hemophagocytic syndrome, and drug-induced myelosuppression (30, 33, 34). In this study, 89 patients (84%) developed ARDS, and some of these patients may have developed thrombocytopenia as a result of decreased platelet release from the pulmonary circulation and increased platelet consumption due to microthrombi.

Recent studies have reported that thrombocytopenia is related to COVID-19 patients’ worse laboratory and clinical outcomes (35–38). To our knowledge, this is the first report of an association between thrombocytopenia and secondary bacterial infection in patients with COVID-19. Thrombocytopenia caused by COVID-19 has been confirmed to increase inflammatory markers, and the incidence of disseminated intravascular coagulation (DIC), ARDS, ICU admission, and mortality (36, 38). Thrombocytopenia can reportedly be used as a prognostic indicator of severity and mortality of COVID-19 (35, 37). There are several plausible explanations of the mechanism whereby thrombocytopenia leads to poor clinical outcomes. The first is immunothrombosis. According to previous studies, the platelets of patients with COVID-19 tend to aggregate with T cells, monocytes, and neutrophils, causing immunothrombosis (39–41). In patients with COVID-19, this immune-mediated thrombosis can occur in multiple organs, including the lungs, and is closely related to disease severity and mortality (42). Second, thrombocytopenia increases the permeability of the systemic and pulmonary vessels, which can contribute to the progression of sepsis and ARDS (43, 44). The last explanation is that platelets act as a defense mechanism in the immune response and serve as effector cells. This last explanation may be the mechanism whereby thrombocytopenia acted as a risk factor for bacterial superinfection in our study. Traditionally, platelets have been thought to act only on hemostasis; however, several recent studies have revealed the inflammatory and immune capabilities of platelets. Platelets contain several pro-inflammatory and anti-inflammatory molecules and interact with various types of immune cells by secreting them (45–47). For example, platelets can recruit leukocytes by recognizing intravascular pathogens using functional pattern recognition receptors such as toll-like receptors (TLRs) located on the surface and secreting various chemokines (48). TLR4-dependent platelet–neutrophil interaction is responsible for the removal of intravascular bacteria by forming neutrophil extracellular traps (NETs) during Gram-negative bacterial infections (49). Additionally, in the murine model, platelet glycoprotein Ib (GPIb), also known as CD42, recognizes vascular pathogens and presents them to macrophages and dendritic cells (50). The mechanisms whereby platelets are responsible for innate and adaptive immunity is a topic of ongoing research (51). In summary, when critically ill patients with COVID-19 develop thrombocytopenia, the risk of secondary bacterial infection increases because the number of platelets available for defense against bacterial infection decreases. Further studies are required to understand the underlying pathophysiological mechanisms better.

The key findings of this study are as follows: (1) Bacterial superinfection occurred frequently in critically ill patients with COVID-19 pneumonia, leading to worse clinical outcomes. (2) Infections caused by MDR pathogens occurred frequently during ICU stay, even in patients with no evidence of colonization by MDR bacteria on ICU admission. (3) To the best of our knowledge, this study is the first to report that thrombocytopenia, a poor prognostic factor in COVID-19, is also associated with secondary bacterial infection and worse clinical outcomes. Moreover, bacterial superinfections caused by MDR pathogens occurred only in patients with higher SAPS II scores and thrombocytopenia.

However, our study had several limitations. First, it was performed at a single tertiary academic hospital. Therefore, our results may not necessarily be generalizable to other hospital settings. Second, given the retrospective nature of this study, some inadequate or missing data may have affected the outcomes. Finally, although fungal and cytomegalovirus co-infections are known to occur commonly in patients with COVID-19 pneumonia, our center did not routinely screen for them, and serum beta-d-glucan, serum galactomannan, and CMV antigenemia in the blood were not investigated. Therefore, we focused on the results regarding the presence of bacterial superinfections and MDR pathogens.

## 5. Conclusion

Bacterial superinfections were common in critically ill patients with severe COVID-19 pneumonia, and more than one-third of these infections were caused by MDR pathogens. Moreover, patients with bacterial superinfections had worse clinical outcomes, including fewer ventilator-free days and longer ICU stays and hospital stays after ICU admission than those without bacterial superinfections. Higher SAPS II scores and thrombocytopenia were independent risk factors for the development of bacterial superinfection. Patients with these risk factors had a significantly higher incidence of bacterial superinfection and worse clinical outcomes than those without these risk factors. Because critically ill patients with severe COVID-19 pneumonia often require prolonged mechanical ventilation, they should be actively monitored for the development of bacterial superinfections.

## Data availability statement

The raw data supporting the conclusions of this article will be made available by the authors, without undue reservation.

## Ethics statement

The studies involving human participants were reviewed and approved by Institutional review boards of Seoul National University Hospital. Written informed consent for participation was not required for this study in accordance with the national legislation and the institutional requirements.

## Author contributions

SY, JL, S-ML, and HL: conceptualization, methodology, and writing – review and editing. SY and HL: data curation. SY: formal analysis. HL: investigation. HL: project administration. SY: writing – original draft. All authors contributed to the article and approved the submitted version.

## Funding

This research was supported by a grant of the Korea Health Technology R&D Project through the Korea Health Industry Development Institute (KHIDI), funded by the Ministry of Health and Welfare, Republic of Korea (grant number: HI21C1074). The funder had no role in the design of the study, the collection and analysis of the data, or the preparation of the manuscript.

## Conflict of interest

The authors declare that the research was conducted in the absence of any commercial or financial relationships that could be construed as a potential conflict of interest.

## Publisher’s note

All claims expressed in this article are solely those of the authors and do not necessarily represent those of their affiliated organizations, or those of the publisher, the editors and the reviewers. Any product that may be evaluated in this article, or claim that may be made by its manufacturer, is not guaranteed or endorsed by the publisher.

## References

[1] WHO Coronavirus (COVID-19) dashboard. Available at: https://covid19.who.int/ (Accessed June 20, 2022)

[2] Ayaz A Arshad A Malik H Ali H Hussain E Jamil B. Risk factors for intensive care unit admission and mortality in hospitalized COVID-19 patients. Acute Crit Care. (2020) 35:249–54. doi: 10.4266/acc.2020.00381, PMID: 33172229 PMC7808857

[3] Huang C Wang Y Li X Ren L Zhao J Hu Y. Clinical features of patients infected with 2019 novel coronavirus in Wuhan. China Lancet. (2020) 395:497–506. doi: 10.1016/S0140-6736(20)30183-5, PMID: 31986264 PMC7159299

[4] Tomazini BM Maia IS Cavalcanti AB Berwanger O Rosa RG Veiga VC. Effect of dexamethasone on days alive and ventilator-free in patients with moderate or severe acute respiratory distress syndrome and COVID-19: the CoDEX randomized clinical trial. JAMA. (2020) 324:1307–16. doi: 10.1001/jama.2020.17021, PMID: 32876695 PMC7489411

[5] Villar J Ferrando C Martínez D Ambrós A Muñoz T Soler JA. Dexamethasone treatment for the acute respiratory distress syndrome: a multicentre, randomised controlled trial. Lancet Respir Med. (2020) 8:267–76. doi: 10.1016/S2213-2600(19)30417-5, PMID: 32043986

[6] Group RC Horby P Lim WS Emberson JR Mafham M Bell JL. Dexamethasone in Hospitalized Patients with Covid-19. N Engl J Med. (2021) 384:693–704. doi: 10.1056/NEJMoa2021436 32678530 PMC7383595

[7] Bernard GR Luce JM Sprung CL Rinaldo JE Tate RM Sibbald WJ. High-dose corticosteroids in patients with the adult respiratory distress syndrome. N Engl J Med. (1987) 317:1565–70. doi: 10.1056/NEJM198712173172504 3317054

[8] Qin C Zhou L Hu Z Zhang S Yang S Tao Y. Dysregulation of immune response in patients with coronavirus 2019 (COVID-19) in Wuhan. China Clin Infect Dis. (2020) 71:762–8. doi: 10.1093/cid/ciaa248, PMID: 32161940 PMC7108125

[9] Abelenda-Alonso G Rombauts A Gudiol C Meije Y Ortega L Clemente M. Influenza and bacterial coinfection in adults with community-acquired pneumonia admitted to conventional wards: risk factors, clinical features, and outcomes. Open Forum Infect Dis. (2020) 7:ofaa066. doi: 10.1093/ofid/ofaa066, PMID: 32206675 PMC7081386

[10] Liu Y Ling L Wong SH Wang MH Fitzgerald JR Zou X. Outcomes of respiratory viral-bacterial co-infection in adult hospitalized patients. EClinicalMedicine. (2021) 37:100955. doi: 10.1016/j.eclinm.2021.100955, PMID: 34386745 PMC8343259

[11] Cataño-Correa JC Cardona-Arias JA Porras Mancilla JP García MT. Bacterial superinfection in adults with COVID-19 hospitalized in two clinics in Medellín-Colombia, 2020. PLoS One. (2021) 16:e0254671. doi: 10.1371/journal.pone.0254671, PMID: 34255801 PMC8277025

[12] Garcia-Vidal C Sanjuan G Moreno-García E Puerta-Alcalde P Garcia-Pouton N Chumbita M. Incidence of co-infections and superinfections in hospitalized patients with COVID-19: a retrospective cohort study. Clin Microbiol Infect. (2021) 27:83–8. doi: 10.1016/j.cmi.2020.07.041, PMID: 32745596 PMC7836762

[13] Langford BJ So M Raybardhan S Leung V Westwood D MacFadden DR. Bacterial co-infection and secondary infection in patients with COVID-19: a living rapid review and meta-analysis. Clin Microbiol Infect. (2020) 26:1622–9. doi: 10.1016/j.cmi.2020.07.016, PMID: 32711058 PMC7832079

[14] Omoush SA Alzyoud JAM. The prevalence and impact of coinfection and superinfection on the severity and outcome of COVID-19 infection: an updated literature review. Pathogens. (2022) 11:445. doi: 10.3390/pathogens11040445, PMID: 35456120 PMC9027948

[15] Ripa M Galli L Poli A Oltolini C Spagnuolo V Mastrangelo A. Secondary infections in patients hospitalized with COVID-19: incidence and predictive factors. Clin Microbiol Infect. (2021) 27:451–7. doi: 10.1016/j.cmi.2020.10.021, PMID: 33223114 PMC7584496

[16] Alhazzani W Evans L Alshamsi F Møller MH Ostermann M Prescott HC. Surviving sepsis campaign guidelines on the management of adults with coronavirus disease 2019 (COVID-19) in the ICU: first update. Crit Care Med. (2021) 49:e219–34. doi: 10.1097/CCM.0000000000004899, PMID: 33555780

[17] Therapeutics and COVID-19. Living guideline, 22 April 2022. Geneva: World Health Organization; 2022 (WHO/2019-nCoV/therapeutics/2022.3). Licence: CC BY-NC-SA 3.0 IGO (2022).

[18] Azoulay E Russell L Van de Louw A Metaxa V Bauer P Povoa P. Diagnosis of severe respiratory infections in immunocompromised patients. Intensive Care Med. (2020) 46:298–314. doi: 10.1007/s00134-019-05906-5, PMID: 32034433 PMC7080052

[19] Ramirez JA Musher DM Evans SE dela Cruz C Crothers KA Hage CA. Treatment of community-acquired pneumonia in immunocompromised adults: a consensus statement regarding initial strategies. Chest. (2020) 158:1896–911. doi: 10.1016/j.chest.2020.05.598, PMID: 32561442 PMC7297164

[20] Rubin LG Levin MJ Ljungman P Davies EG Avery R Tomblyn M. 2013 IDSA clinical practice guideline for vaccination of the immunocompromised host. Clin Infect Dis. (2014) 58:309–18. doi: 10.1093/cid/cit816, PMID: 24421306

[21] Hultström M Hellkvist O Covaciu L Fredén F Frithiof R Lipcsey M. Limitations of the ARDS criteria during high-flow oxygen or non-invasive ventilation: evidence from critically ill COVID-19 patients. Crit Care. (2022) 26:55. doi: 10.1186/s13054-022-03933-1, PMID: 35255949 PMC8899791

[22] Matthay MA Thompson BT Ware LB. The Berlin definition of acute respiratory distress syndrome: should patients receiving high-flow nasal oxygen be included? Lancet Respir Med. (2021) 9:933–6. doi: 10.1016/S2213-2600(21)00105-3, PMID: 33915103 PMC8075801

[23] Magiorakos AP Srinivasan A Carey RB Carmeli Y Falagas ME Giske CG. Multidrug-resistant, extensively drug-resistant and pandrug-resistant bacteria: an international expert proposal for interim standard definitions for acquired resistance. Clin Microbiol Infect. (2012) 18:268–81. doi: 10.1111/j.1469-0691.2011.03570.x, PMID: 21793988

[24] Wolfensberger A Kuster SP Marchesi M Zbinden R Hombach M. The effect of varying multidrug-resistence (MDR) definitions on rates of MDR gram-negative rods. Antimicrob Resist Infect Control. (2019) 8:193. doi: 10.1186/s13756-019-0614-3, PMID: 31798839 PMC6883537

[25] Bardi T Pintado V Gomez-Rojo M Escudero-Sanchez R Azzam Lopez A Diez-Remesal Y. Nosocomial infections associated to COVID-19 in the intensive care unit: clinical characteristics and outcome. Eur J Clin Microbiol Infect Dis. (2021) 40:495–502. doi: 10.1007/s10096-020-04142-w, PMID: 33389263 PMC7778834

[26] Buetti N Ruckly S de Montmollin E Reignier J Terzi N Cohen Y. COVID-19 increased the risk of ICU-acquired bloodstream infections: a case-cohort study from the multicentric OUTCOMEREA network. Intensive Care Med. (2021) 47:180–7. doi: 10.1007/s00134-021-06346-w, PMID: 33506379 PMC7839935

[27] Peduzzi P Concato J Kemper E Holford TR Feinstein AR. A simulation study of the number of events per variable in logistic regression analysis. J Clin Epidemiol. (1996) 49:1373–9. doi: 10.1016/s0895-4356(96)00236-3, PMID: 8970487

[28] Pickens CO Gao CA Cuttica MJ Smith SB Pesce LL Grant RA. Bacterial superinfection pneumonia in patients mechanically ventilated for COVID-19 pneumonia. Am J Respir Crit Care Med. (2021) 204:921–32. doi: 10.1164/rccm.202106-1354OC, PMID: 34409924 PMC8534629

[29] Pourajam S Kalantari E Talebzadeh H Mellali H Sami R Soltaninejad F. Secondary bacterial infection and clinical characteristics in patients with COVID-19 admitted to two intensive care units of an academic Hospital in Iran during the first wave of the pandemic. Front Cell Infect Microbiol. (2022) 12:784130. doi: 10.3389/fcimb.2022.784130, PMID: 35281440 PMC8904895

[30] Xu P Zhou Q Xu J. Mechanism of thrombocytopenia in COVID-19 patients. Ann Hematol. (2020) 99:1205–8. doi: 10.1007/s00277-020-04019-0, PMID: 32296910 PMC7156897

[31] Jurek T Rorat M Szleszkowski L Tokarski M Pielka I Malodobra-Mazur M. SARS-CoV-2 viral RNA is detected in the bone marrow in post-mortem samples using RT-LAMP. Diagnostics (Basel). (2022) 12:515. doi: 10.3390/diagnostics12020515, PMID: 35204605 PMC8870857

[32] Lefrançais E Ortiz-Muñoz G Caudrillier A Mallavia B Liu F Sayah DM. The lung is a site of platelet biogenesis and a reservoir for haematopoietic progenitors. Nature. (2017) 544:105–9. doi: 10.1038/nature21706, PMID: 28329764 PMC5663284

[33] Bomhof G Mutsaers P Leebeek FWG te Boekhorst PAW Hofland J Croles FN. COVID-19-associated immune thrombocytopenia. Br J Haematol. (2020) 190:e61–4. doi: 10.1111/bjh.16850, PMID: 32420612 PMC7276755

[34] Zhang C Shi L Wang FS. Liver injury in COVID-19: management and challenges. Lancet Gastroenterol Hepatol. (2020) 5:428–30. doi: 10.1016/S2468-1253(20)30057-1, PMID: 32145190 PMC7129165

[35] Liao D Zhou F Luo L Xu M Wang H Xia J. Haematological characteristics and risk factors in the classification and prognosis evaluation of COVID-19: a retrospective cohort study. Lancet Haematol. (2020) 7:e671–8. doi: 10.1016/S2352-3026(20)30217-9, PMID: 32659214 PMC7351397

[36] Simsek M Yildirim F Karaman I Dural HI. Hematological manifestations of COVID-19 acute respiratory distress syndrome patients and the impact of thrombocytopenia on disease outcomes: a retrospective study. Int J Crit Illn Inj Sci. (2022) 12:95–100. doi: 10.4103/ijciis.ijciis_96_21, PMID: 35845123 PMC9285121

[37] Tang N Li D Wang X Sun Z. Abnormal coagulation parameters are associated with poor prognosis in patients with novel coronavirus pneumonia. J Thromb Haemost. (2020) 18:844–7. doi: 10.1111/jth.14768, PMID: 32073213 PMC7166509

[38] Zong X Gu Y Yu H Li Z Wang Y. Thrombocytopenia is associated with COVID-19 severity and outcome: an updated meta-analysis of 5637 patients with multiple outcomes. Lab Med. (2021) 52:10–5. doi: 10.1093/labmed/lmaa067, PMID: 32929506 PMC7543465

[39] Manne BK Denorme F Middleton EA Portier I Rowley JW Stubben C. Platelet gene expression and function in patients with COVID-19. Blood. (2020) 136:1317–29. doi: 10.1182/blood.2020007214, PMID: 32573711 PMC7483430

[40] Middleton EA He XY Denorme F Campbell RA Ng D Salvatore SP. Neutrophil extracellular traps contribute to immunothrombosis in COVID-19 acute respiratory distress syndrome. Blood. (2020) 136:1169–79. doi: 10.1182/blood.2020007008, PMID: 32597954 PMC7472714

[41] Hottz ED Azevedo-Quintanilha IG Palhinha L Teixeira L Barreto EA Pão CRR. Platelet activation and platelet-monocyte aggregate formation trigger tissue factor expression in patients with severe COVID-19. Blood. (2020) 136:1330–41. doi: 10.1182/blood.2020007252, PMID: 32678428 PMC7483437

[42] Loo J Spittle DA Newnham M. COVID-19, immunothrombosis and venous thromboembolism: biological mechanisms. Thorax. (2021) 76:412–20. doi: 10.1136/thoraxjnl-2020-216243, PMID: 33408195

[43] Ho-Tin-Noe B Demers M Wagner DD. How platelets safeguard vascular integrity. J Thromb Haemost. (2011) 9:56–65. doi: 10.1111/j.1538-7836.2011.04317.x, PMID: 21781242 PMC3229170

[44] Weyrich AS Zimmerman GA. Platelets in lung biology. Annu Rev Physiol. (2013) 75:569–91. doi: 10.1146/annurev-physiol-030212-183752, PMID: 23043249 PMC3670819

[45] Semple JW Italiano JE Jr Freedman J. Platelets and the immune continuum. Nat Rev Immunol. (2011) 11:264–74. doi: 10.1038/nri2956, PMID: 21436837

[46] Kapur R Zufferey A Boilard E Semple JW. Nouvelle cuisine: platelets served with inflammation. J Immunol. (2015) 194:5579–87. doi: 10.4049/jimmunol.1500259, PMID: 26048965

[47] Li C Li J Li Y Lang S Yougbare I Zhu G. Crosstalk between platelets and the immune system: old systems with new discoveries. Adv Hematol. (2012) 2012:384685:1–14. doi: 10.1155/2012/384685, PMID: 23008717 PMC3447344

[48] Gaertner F Massberg S. Patrolling the vascular borders: platelets in immunity to infection and cancer. Nat Rev Immunol. (2019) 19:747–60. doi: 10.1038/s41577-019-0202-z, PMID: 31409920

[49] McDonald B Urrutia R Yipp BG Jenne CN Kubes P. Intravascular neutrophil extracellular traps capture bacteria from the bloodstream during sepsis. Cell Host Microbe. (2012) 12:324–33. doi: 10.1016/j.chom.2012.06.011, PMID: 22980329

[50] Wong CH Jenne CN Petri B Chrobok NL Kubes P. Nucleation of platelets with blood-borne pathogens on Kupffer cells precedes other innate immunity and contributes to bacterial clearance. Nat Immunol. (2013) 14:785–92. doi: 10.1038/ni.2631, PMID: 23770641 PMC4972575

[51] Li C Li J Ni H. Crosstalk between platelets and microbial pathogens. Front Immunol. (2020) 11:1962. doi: 10.3389/fimmu.2020.01962, PMID: 32849656 PMC7426443

